# S. Bagaeen and O. Uduku: Beyond gated communities: Routledge, New York, 2015, 246 pp., ISSN:978-0-425-74824-7

**DOI:** 10.1007/s10901-016-9504-7

**Published:** 2016-02-01

**Authors:** Tony Manzi

**Affiliations:** University of Westminster, London , UK

How much more can be added to the discussion on gated communities that has not been addressed in earlier studies? In other words what lies behind gated communities? This book follows a previous collection, published in 2010—*Gated Communities: Social Sustainability in Contemporary and Historical Gated Developments*—also edited by Bagaeen and Uduku. Like the previous edition, the current volume adopts a global perspective in the analysis of gated communities, but the intention is to provide a more critical account, introducing the key notion of gating as a variable, defined by Sassen (in the foreword) as ‘thick, localised interventions by diverse actors, who can be local or global, good or bad’ (p. xviii). What is interesting about this approach is that the concept is not limited to the stereotypical view of gating as a local-level phenomenon confined to the most affluent groups. This enables a richer analysis of a variety of processes, and provides a wide-ranging, theoretically informed and critical set of studies.

The editors have brought together a highly diverse set of case studies and this diversity is both a strength and weakness. It offers a useful and fascinating insight into different experiences of gating, demonstrating that these processes can take very different forms. It is also able to provide new perspectives on patterns of socio-spatial segregation, exclusion and marginality. However, the weakness is (as with all collections of this type) that at times the chapters lack coherence. The introduction is brief; an extended chapter here could have explained core themes and highlight similarities and contrasts between the cases more effectively. Important ideas are raised about conflict, soft boundaries and networks of influence and affluence; these are developed in some but not all chapters. Nevertheless, the core themes are clearly explained at the outset and Bagaeen provides a useful conceptual analysis of different approaches to the phenomenon of urban gating. More discussion of how the studies were collected would be useful—why for example were three chapters on South Africa thought necessary?

The most successful chapters are those which shed new light on gating processes. The chapter on ‘Occupy London’ (by Robinson) at first seems incongruous in a book about gated communities, but it does provide a thoughtful conceptual analysis of the differences between open and closed forms of gating. Duca’s chapter on gating in South Africa provides insight into how gated communities are embedded into wider social systems, based on (rarely conducted in this field) ethnographic study (although Fayel’s chapter on Israel in this collection provides another example). It is these relational and interactive features which provide the most original and thoughtful sections of the book. For example Duca sees gated communities as ‘constitutive of systems of governance’, ‘distinct sites of a particular form of disengagement’ and more than simply ‘pockets of wealth’ (p. 50). The chapter on Ireland (by Kenna et al.) provides a useful illustration of how small-scale examples of gating, used to address anti-social behaviour, can have much wider social consequences. Rosen and Walk’s chapter on ‘condo-ism’ as a way of life (based on a Toronto case study) offers insight into how tenure types can alter social relations, and how a shift towards densification has adopted privatised (and securitised) forms.

The strength of other chapters (including discussions of gating in South Korea, Thailand, South Africa, Israel, Mexico and Chile) is their focus on relationships between the state and civil society and changing patterns of structure and agency. The chapter on South Korea (by Kim) focuses on contradictions of the state whilst Spoctor (on South Africa) shows how geographies of gating can alter social relations and networks of power. Referring to Baumgartner’s (1988) notion of ‘moral minimalism’ or a ‘culture of avoidance’ in middle-class suburban neighbourhoods, Fayel shows (in a study of Israel) how transference of risk can increase fear and insecurity in the wider community. Milian Avila and Guenet adopt a highly critical (and possibly overstated) analysis of gating in Mexico, arguing that the ‘perverse relationships’ engendered by gated communities can ‘threaten the future of global society as a whole’ (p. 199). Some chapters contain some highly questionable assumptions, for example in discussion of Thailand, Suwannasang contends that a dramatic increase in land value leads to better redistribution of income, social interaction and quality of life (p. 109). Whilst an analysis of the morphology of gating is valuable, I was unconvinced by the metaphor (in Nel and Landman's chapter) of a gated community as a ‘tree’. The final chapter considers ‘new geographies of power’ through photographic representation of a corporate mining town in Chile, but the images at the end would benefit from further analysis.

A curious omission is that the term ‘social sustainability’ was given so little attention in this collection (it only appears once in the index)—perhaps the editors considered that this issue had been comprehensively considered in their earlier contribution? It would also have been helpful to include more discussion of changes in approach since 2010. The work also lacks a conclusion which might have drawn the various issues together and point to emerging trends more clearly. For example what can the various chapters tell us about how conflict, isolation and fear operate in inner city and suburban areas within the landscape of the neoliberal city? Nevertheless, the focus on relational and interactive aspects between gating and other parts of the city does provide an interesting new perspective on a topic, which (as Sassen notes in the foreword) might otherwise have been considered as a little tired. As the contemporary refugee crisis in Europe so clearly illustrates, the fact that countries (such as Hungary) can construct gates across their borders, shows how the phenomenon of gating continues to have a powerful resonance and the conceptual, global analysis provided in this collection helps in understanding how such processes operate.
